# A Sterile, Injectable, and Robust Sericin Hydrogel Prepared by Degraded Sericin

**DOI:** 10.3390/gels9120948

**Published:** 2023-12-03

**Authors:** Yeshun Zhang, Susu Wang, Yurong Li, Xiang Li, Zhanyan Du, Siyu Liu, Yushuo Song, Yanyan Li, Guozheng Zhang

**Affiliations:** 1 School of Biotechnology, Jiangsu University of Science and Technology, Zhenjiang 212100, China; 209310023@stu.just.edu.cn (S.W.); yrli@just.edu.cn (Y.L.); 221211801104@stu.just.edu.cn (X.L.); 221111802105@stu.just.edu.cn (Z.D.); 202211802107@stu.just.edu.cn (S.L.); 212211802204@stu.just.edu.cn (Y.L.); zgzsri@just.edu.cn (G.Z.); 2 Key Laboratory of Silkworm and Mulberry Genetic Improvement, Ministry of Agriculture and Rural Affairs, Sericultural Research Institute, Chinese Academy of Agricultural Sciences, Zhenjiang 212100, China; 3 Zhenjiang Zhongnong Biotechnology Co., Ltd., Zhenjiang 212121, China

**Keywords:** hydrogel, high temperature and pressure, degraded sericin, robustness

## Abstract

The application of sericin hydrogels is limited mainly due to their poor mechanical strength, tendency to be brittle and inconvenient sterilization. To address these challenges, a sericin hydrogel exhibiting outstanding physical and chemical properties along with cytocompatibility was prepared through crosslinking genipin with degraded sericin extracted from fibroin deficient silkworm cocoons by the high temperature and pressure method. Our reported sericin hydrogels possess good elasticity, injectability, and robust behaviors. The 8% sericin hydrogel can smoothly pass through a 16 G needle. While the 12% sericin hydrogel remains intact until its compression ratio reaches 70%, accompanied by a compression strength of 674 kPa. 12% sericin hydrogel produce a maximum stretch of 740%, with breaking strength and tensile modulus of 375 kPa and 477 kPa respectively. Besides that, the hydrogel system demonstrated remarkable cell-adhesive capabilities, effectively promoting cell attachment and, proliferation. Moreover, the swelling and degradation behaviors of the hydrogels are pH responsiveness. Sericin hydrogel releases drugs in a sustained manner. Furthermore, this study addresses the challenge of sterilizing sericin hydrogels (sterilization will inevitably lead to the destruction of their structures). In addition, it challenges the prior notion that sericin extracted under high temperature and pressure is difficult to directly cross-linked into a stable hydrogel. This developed hydrogel system in this study holds promise to be a new multifunctional platform expanding the application area scope of sericin.

## 1. Introduction

Polymer hydrogels, characterized by with three-dimensional network structure containing a large amount of water, have garnered persistent attention in biomedical fields by acting as multifunctional platforms for tissue engineering [1], drug delivery [2], cell modulation [3], optical devices [4], and biosensors [5]. Hydrogels formed by natural biological materials were favored in the biomedical field due to their excellent biocompatibility and bioactivities [6]. Recently, sericin, a natural non-immunogenic protein with multiple biological activities, has received great attention and is considered as a new promising candidate for fabricating hydrogel [1].

Sericin, a bioactive protein containing many polypeptides with varying molecular weights, is produced from the middle silk gland of silkworms. Sericin is abundance in resources and yield. It is estimated that there are more than thousands of silkworm strains and the annual output of sericin is over 50,000 tons in the world [7,8]. Silkworms are classified into two categories: wild-type or fibroin-deficient mutant [9,10]. The cocoons of wild-type silkworms contain high amounts of fibroin (approximately 75%) [11]. Currently, the existing extracting methods for sericin from wild-type cocoons involve harsh degumming, resulting in the degradation of sericin and the formation of a highly water-soluble structure. This degraded sericin is difficult to form stable hydrogel network [1]. Generally, the degraded sericin is mixed into other polymers (polyacrylamide [12], poly(*N*-isopropylacrylamide) [13], chitosan [14,15,16], Poly(vinyl alcohol) [17,18], sodium alginate [19,20], agar [21], poly(ethylene glycol) dimethacrylate [21], collagen [22], gelatin [23]) hydrogel systems to improve their physical-chemical properties or biological activities.

For a long time, it is a challenge to fabricate pure sericin hydrogels with stable networks. Until the early 21st century, the fibroin-deficient mutant cocoons (e.g., Sericin hope, *140 Nd-s*) without fibroin were developed to obtain intact and non-degraded sericin by a gentle extraction method (for example, LiBr) [8,24]. This type of sericin was prone to form stable hydrogel networks by chemical crosslinking agents or physical factors. A series of sericin hydrogels were successfully developed based on such sericin by treating using glutaraldehyde [8], genipin [25], and physical sonication [2]. However, it is difficult to obtain high concentration of native sericin from cocoons through the mild extracting process at present. These sericin hydrogels are poor in elastic modulus and inconvenient for sterilization (the structure of sericin hydrogels would be unavoidably destroyed under existing sterilization methods [26]), resulting in seriously restricting their practical applications. To address these problems, the high temperature and pressure method is employed to extract high-content sericin solution from fibroin-deficient cocoons powder for fabricating a sterile hydrogel with high hardness, avoiding the subsequent complex sterilization processes. Depressingly, this hydrogel is brittle and fragile [26]. Furthermore, we fabricated a robust sericin hydrogel based on a high concentration native sericin isolated from fibroin-deficient silkworm bodies [27]. But some problems in this hydrogel system still exist, including laborious sericin extraction processes and inconvenient sterilization, thus limiting its application. Now, it is still a challenge to fabricate a sterile robust sericin hydrogel. 

In this work, a new sericin hydrogel with excellent physical-chemical properties and cytocompatibility was fabricated by a simple process. Once the sericin gelling reaction system is placed in a bio-clean environment, a sterile and robust sericin hydrogel can be fabricated directly. Our new hydrogel system overcomes the problem that sericin hydrogels are difficult to be sterilized. Moreover, this sericin hydrogel possesses good elasticity, injectability, and robust behaviors. 

## 2. Results and Discussion

### 2.1. Synthesis and Mechanism of Sericin Hydrogels

**Figure 1:**
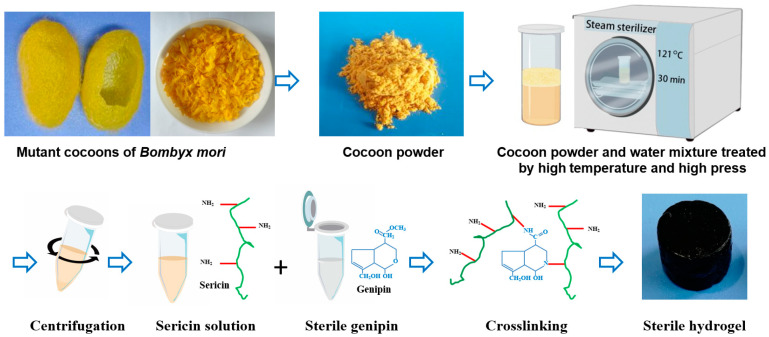
The isolation, purification, and fabrication of the sterile and robust sericin hydrogel. Fibroin-deficient cocoons were cut into powder by a grinder with high-speed blades. Next, the powder-ultra water mixture was treated using high temperature and pressure. The soup was centrifuged to collect the supernatant of the sericin solution. Then, the sericin solution was crosslinked with sterile genipin dried from genipin solution that was sterilized by filtration using 0.22 μm membrane, forming a robust sterile sericin hydrogels with dark blue.

**Figure 2:**
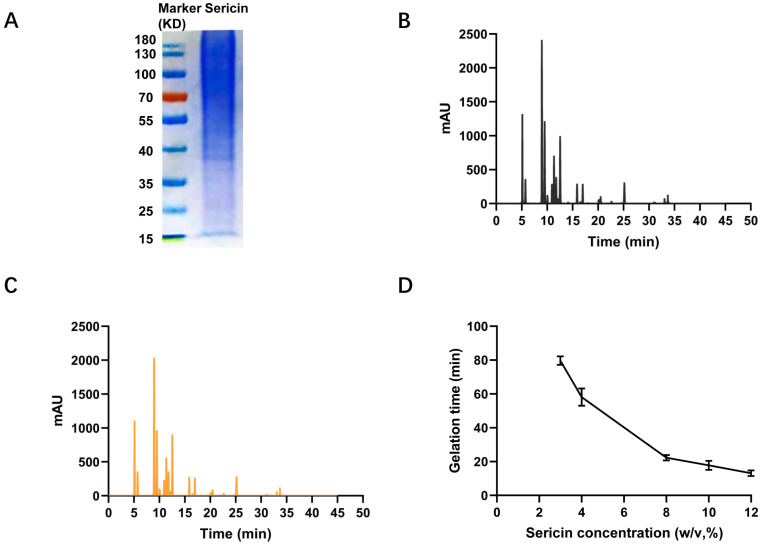
(**A**) The protein profile of sericin (left lane is marker and right lane is sericin). HPLC chromatograms of sericin isolated from *Jingsong A* wild-type silkworm cocoons (**B**) and *180 Nd-s* fibroin-deficient silkworm cocoons (**C**). (**D**) The relationship between sericin concentration and gelation time.

The sericin hydrogel was formed by sericin which was isolated from yellow fibroin-deficient mutant silkworm cocoons (*180 Nd-s*) using a high temperature and pressure method. The detailed preparation process of sericin hydrogel was described in Figure 1. A diffused electrophoretogram with vague bands ranging from 10~ > 180 kDa revealed sericin was degraded seriously (Figure 2A) which was consistent with previously reported [8]. Interestingly, in our previous study, we revealed that the solubility of sericin of fibroin-deficient mutant silkworm cocoons in hot water is far higher than that of wild-type silkworm cocoons [26]. Only a small amount of sericin protein can be dissolved in hot water in the wild-type silkworm cocoons [28]. Intriguingly, the sericin in the cocoon powder could be almost completely dissolved in our study. The results indicated that the compositions of sericin solution in this study are different from that extracted from wild-type silkworm cocoons. Previous reports argued that the sericin isolated from silkworm cocoons by harsh extracting methods, including high temperature and pressure, is hard to crosslink into stable sericin hydrogel due to its high degradation and water solubility [1]. To be excited, we found such degraded sericin could form a robust hydrogel with excellent elasticity (Supplementary Videos S1 and S2) in the presence of genipin. Why the degraded sericin can be crosslinked into a stable hydrogel by genipin? As we know, genipin is a popular and biocompatible crosslinker for fabricating hydrogels or scaffolds by reacting with free amino groups of polysaccharides or peptides [29]. To address this confusion, we determined the content of free amino end group and the amino acid composition of the sericin. The results showed that the content of free amino end groups in the sericin isolated from *180 Nd-s* was as high as 12 times that in the sericin extracted from wild-type silkworms (Jingsong A) (Table 1). Sericin with higher content of free amino end groups provides more opportunities to crosslink with genipin. Moreover, the higher content of free amino end groups would raise the crosslink density between sericin and genipin. However, there is little difference in amino acid composition of sericins between the wild-type silkworm cocoons and fibroin-deficient mutant silkworm cocoons (Table 1, Figure 2B,C).

### 2.2. Gelation Kinetics of Sericin Hydrogels

The influence of sericin concentrations on the gelation rate of sericin hydrogels was investigated. The results in Figure 2D revealed that the gelation time was shorted as the sericin concentration increased. The gelation times can be adjusted from 13 to 80 min on the basis of the sericin concentration.

### 2.3. The Micro-Architecture of Sericin Hydrogel

**Figure 3:**
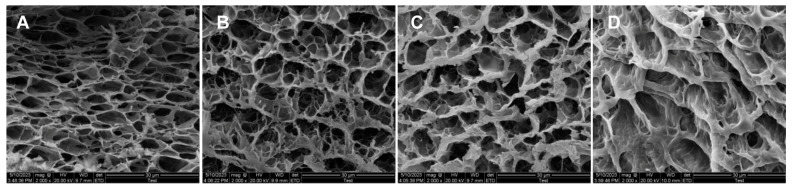
SEM of freeze-dried sericin hydrogels formed by sericin solutions with different concentrations. (**A**) 4% sericin hydrogel, (**B**) 8% sericin hydrogel, (**C**) 10% sericin hydrogel, (**D**) 12% hydrogel.

The micro-architecture of lyophilized sericin hydrogels was observed by scanning electron microscopy (SEM). We analyzed the microstructure of 4%, 8%, 10%, and 12% sericin hydrogels (Figure 3), followed by the calculation of pore size, porosity and pore wall thickness. The results showed that the pore size of these hydrogel scaffolds is distributed between 4.9 μm to 7.1 μm. Porosity was decreased as the concentrations of sericin solutions was increased. The porosities of 4%, 8%, 10%, and 12% sericin hydrogel are 79%, 70%, 67%, and 66%. respectively (Table 2). It is interesting that pore wall thickness of sericin hydrogel is increased with the concentration of sericin solution increased.

### 2.4. Swelling Characteristics of the Sericin Hydrogels

**Figure 4:**
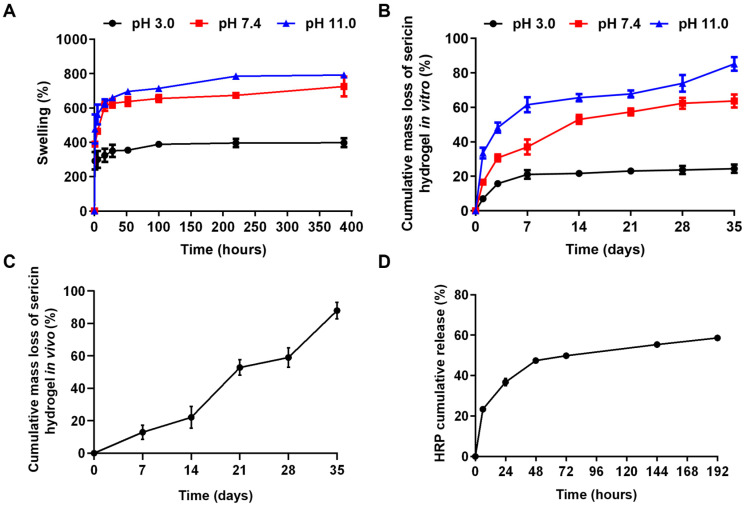
(**A**) Swelling behaviors of the sericin hydrogel in PBS with different pH (**B**,**C**). Degradability dynamics of the sericin hydrogel in vitro (in PBS with different pH) or in vivo. (**D**) The HRP release behavior from the sericin hydrogel in the in the PBS with pH 7.4 at 37 °C.

The swelling properties of hydrogels suggest the capacity to absorb water, which is vital as it affects many aspects of a hydrogel (such as absorb water, volume and so on) [8,30]. Usually, the water absorption of hydrogels can be indicated by swelling properties. To characterize the swelling properties of sericin hydrogels, we determined the swelling dynamics of lyophilized sericin hydrogel at different pH (pH 3.0, pH 7.4, pH 11.0). The results in Figure 4A exhibited the expansibility of 8% sericin hydrogel under different pH conditions. During the first 12 h, the burst swelling appeared no matter what kind of pH conditions. The reason for this phenomenon is that abundant hydrophilic groups contained in sericin are easily combined with water molecules [31]. The first phase was followed by a slowly increasing trend of swelling rate. The peaks of lyophilized sericin hydrogel samples appeared within 100 h under acidic (pH 3.0) or neutral conditions (pH 7.4). Under basic conditions (pH 7.4), the swelling peak occurred relatively late at 225 h. After a period of water absorption and swelling, the swelling ratios of samples reached 400%, 700%, and 800% at pH 3.0, pH 7.4, and pH 11.0, respectively. The results showed that swelling behaviors of the sericin hydrogels are responsive to pH, which is consistent with that of sericin hydrogels in our earlier reports [2,26,27].

### 2.5. Degradability of the Sericin Hydrogels

Controlling the degradation of a hydrogel is important for drug release in vivo application. A degradable hydrogel can release encapsulated bioactive molecules to exert its functions. Here, the in vitro degradation behaviors of sericin hydrogel under different pH conditions were evaluated. As presented in Figure 4B, the degradation of sericin hydrogel was the fastest at alkaline conditions (pH 11.0). The cumulative degradation rate was 85.2% within 3 weeks. While the cumulative degradation rates of sericin hydrogel under neutral conditions (pH 7.4) and acidic conditions (pH 3.0) were 63.7% and 24.5% within 5 weeks, respectively. In agreement with the sericin hydrogels previously reported, the sericin hydrogel had an apparent response to pH. The cause was ascribed to the sericin with pI of 4 and containing abundant acidic amino acids [32]. Compared with self-assembled sericin hydrogel [26], the hydrogel formed by covalent crosslinking in this study has a longer life and better stability in aqueous solution. It shows that covalently crosslinked hydrogels are more stable than self-assembly hydrogels. The reason is that peptide chains in sericin can be covalently bounded by genipin to form more stable compounds. Further, degradation dynamics of the hydrogel in vivo were carried out in C57BL/6 mice. The life span of hydrogel was over 5 weeks in vivo (Figure 4C). The results gave evidence that the sericin hydrogel has a stable network for further evaluating the controlled drug release.

### 2.6. Drug Release of Sericin Hydrogels

The sustained delivery systems could maintain drugs with a sustained release behavior and prolong therapeutic effect, thus improving their utilization [33]. Hydrogels are a type of most important biomaterials for drug delivery. In this study, we employed horseradish peroxidase (HRP) as a model drug to evaluate the drug release kinetics of the sericin hydrogel. Then the drug release profile of HRP from the sericin hydrogel was monitored. As shown in Figure 4D, HRP burst release appeared within the beginning of 6 h, which was consistent with previous reports [2,8]. Then the release rate of HRP slowed down over time. The cumulative release rate of HRP reached approximately 60% within 192 h. The results indicated that sericin hydrogel has great potential in drug delivery.

### 2.7. Secondary Structure and Crystallinity of Sericin Hydrogels

**Figure 5:**
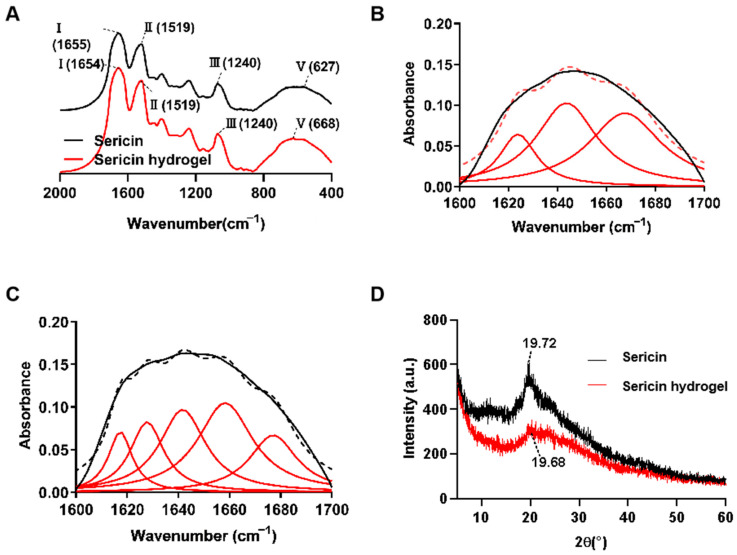
The secondary structure of sericin and sericin hydrogel. (**A**) FTIR spectra of sericin solution and sericin hydrogel. (**B**) Curve-fitted spectra of sericin solution. (**C**) Curve-fitted spectra of sericin hydrogel. (Lorentzian curves (broken black line) were fitted iteratively to the amide I band using the peak positions obtained from the second derivative spectrum as the initial parameters. Prior to data analysis, baseline correction and nine-point Savitzky-Golay smoothing were performed for the amide band.). (**D**) X-ray diffraction curves of sericin solution and sericin hydrogel.

The Fourier transformation infrared spectroscopy (FTIR) and X-ray diffraction (XRD) are used to evaluate the structure of proteins or polypeptides. The structure of a protein was revealed in terms of its characteristic absorption band based on stretching vibrations of the structure repeat. The absorption bands used for analyzing protein secondary structure include amide I, amide II, amide III, and amide V. The amide I (1700–1600 cm^−1^) represents the C=O stretching vibration of peptide bonds. The amide II (1600–1500 cm^−1^) is mainly generated by N–H stretching vibration and C–N stretching vibration of amino acids. The absorption peak of amide III (1330–1220 cm^−1^) mainly comes from C–N stretching vibration coupled with amino acid N–H in-plane bending vibration. The amide V (800–640 cm^−1^) is mainly determined by the N–H stretching vibration outside the plane. Among them, amide I is most sensitive to change in the secondary structure of proteins. Hence, amide I is most useful to analyze the secondary structure of proteins [34,35,36,37,38,39]. Generally, the characteristic absorption peaks of β -folding, random coil and α-helix in the amide I are located at 1630 cm^−1^, 1645 cm^−1^, and 1655 cm^−1^, respectively. In order to clarify the composition of secondary structure in sericin before and after gel, amide Ⅰ of samples was analyzed. As shown in Figure 5A–C, the peak position of the amide I band shifted from 1655 cm^−1^ to 1654 cm^−1^ after gel by comparing the spectra of sericin solution with sericin hydrogel. According to the fitting curve values, the secondary structure of sericin before and after gel formation was shown in Table 3. Notably, both α-helix and β-sheet in sericin were increased greatly after gel, indicating that sericin polypeptides were transformed into more stable networks by crosslinking with genipin [26].

The crystalline structure of sericin was determined by X-ray diffraction. The diffraction peaks of sericin powder and sericin hydrogel were at 19.72° and 19.68° (Figure 5D). This change may be contributed to the conversion of the random coil into β-sheet structure in sericin [26].

### 2.8. Mechanical Stability of Sericin Hydrogels

**Figure 6:**
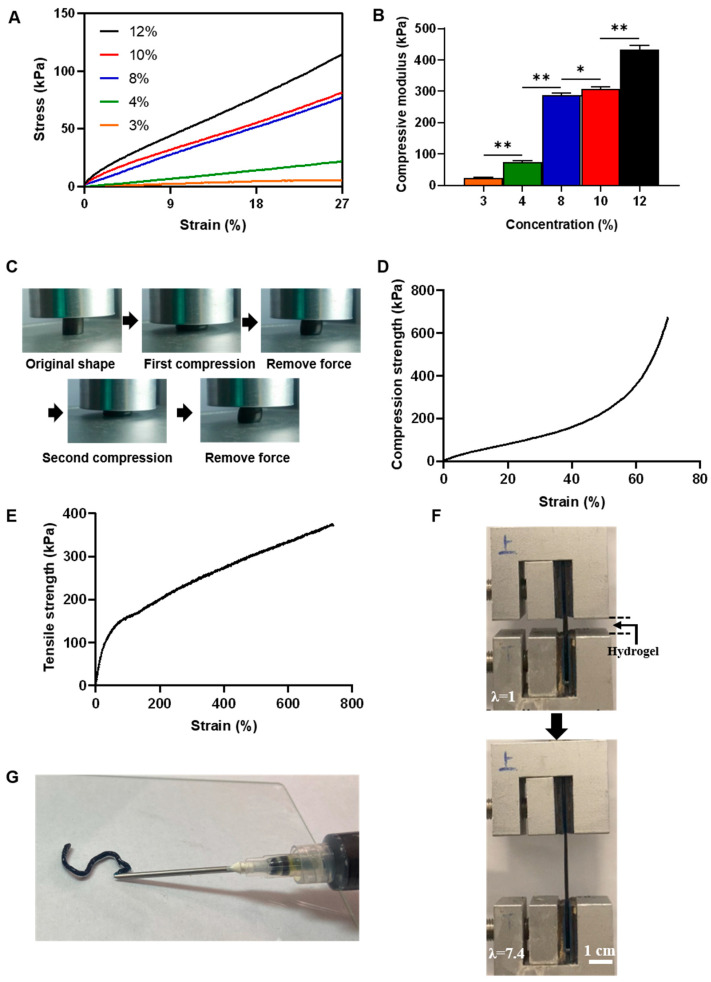
(**A**) The compression stress-strain curves of 3%, 4%, 8%, 10%, and 12% sericin hydrogels. (**B**) Compressive moduli of the sericin hydrogels were fabricated by sericin solutions with different concentrations (*n* = 3 per group; ANOVA test; * *p* < 0.05 ** *p* < 0.01). (**C**) The state of 8% sericin hydrogel under pressure and removal of external force. (**D**) The compression stress-strain curve of 12% sericin hydrogel. (**E**) The tensile stress-strain curve of 12% sericin hydrogel. (**F**) The 12% sericn hydrogel was stretched to 7.4 times its initial length in a tensile test. λ is ratio of the distance between the two clamps when the gel is formed to the distance when the gel is undeformed. All the tests were performed at an extension rate of 5 mm/min. (**G**) The letter “S” were formed by injection.

Mechanical property is one of the key parameters to be evaluated for hydrogel materials. The compression test results as shown in Figure 6A–C and Supplementary Video S1 (right column), S2. The compressive modulus of hydrogel was increase with increasing the concentration of sericin. The compressive moduli of the 3%, 4%, 8%, 10%, and 12% hydrogels were 25, 75, 289, 311, and 434 kPa respectively (Figure 6A,B). High concentration hydrogel with higher compressive moduli may be caused by its lower porosity and thicker pore wall (Figure 3, Table 2). The 8% hydrogel remained intact when its compression ratio was above 50%. Once the external force was removed, the hydrogel sample could recover to over 95.0% of the original (Figure 6C and Supplementary Video S1 (right column)). The 12% sericin hydrogel was unbroken until its compression ratio reached 70%, and accompanied with the compression strength of 674 KPa (Figure 6C and Supplementary Video S2). The above results suggested that the hydrogels formed by degraded sericin own excellent hardness and elasticity. In addition, the maximum stretch of 12% sericin hydrogel could reach 740%, and the breaking strength was 375 kPa in the tensile test. The tensile modulus of the 12% hydrogel is up to 477 kPa (Figure 6B and Supplementary Video S3). These parameters were much higher than that of pure sericin hydrogel formed by sericin isolated from cocoons reported in previous studies [18], indicating that the sericin hydrogel had excellent mechanical properties. 

### 2.9. Syringe-Injectable Property of Sericin Hydrogels

Injectable hydrogel systems are attractive in local drug delivery by providing a 3D hydrogel network within the target tissue capable of sustained release of the chemotherapeutics for increasing the therapeutic index [40,41]. Sericin is widely studied for the synthesis of injectable in situ-forming hydrogels because of ready availability, presence of modifiable functional groups, biocompatibility, and other physiochemical properties [7]. Prior to this study, a few injectable sericin hydrogels were fabricated by low-concentration native sericin isolated from fibroin-deficient cocoons via a mild extracting method (LiBr). In this paper, we propose the problem that whether degraded sericin can be fabricated as an injectable hydrogel. Herein, we used the syringe to evaluate the injectability of the sericin hydrogels formed by sericin with different concentrations. The results showed that the sericin hydrogels formed by sericin solutions at concentration 8% was smoothly through 16 G needles (Figure 6G, Supplementary Video S4). 

### 2.10. Cells Adhesion and Proliferation of the Hydrogels

**Figure 7:**
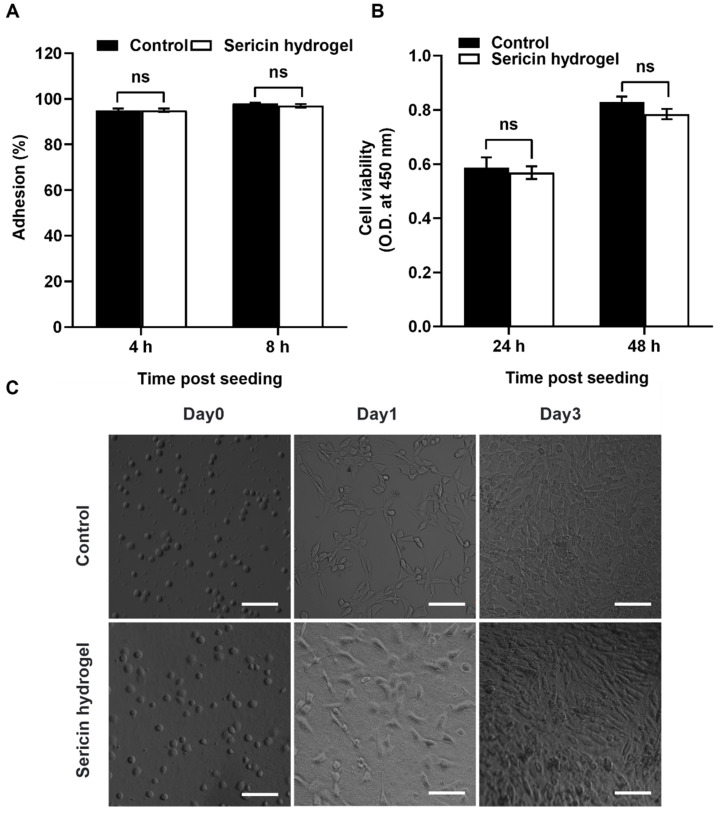
Cytocompatibility evaluation of the sericin hydrogel. (**A**,**B**) Quantification comparison of the cell adhesion (**A**) and cell viability (**B**) between NIH3T3 cells on the culture dishes and the sericin hydrogels. ns, not significant. (**C**) The morphology and proliferation of mouse NIH3T3 fibroblast growing on the culture dishes (upper panel) and the sericin hydrogel (lower panel). Scale bars, 100 μm.

To evaluate the cytocompatibility of the sericin hydrogel, we seeded mouse fibroblast cells (NIH3T3) on the sericin hydrogels. Then, cell adhesion and proliferation of the sericin hydrogel was evaluated. The results showed that there was no significant difference in cell adhesion between sericin hydrogel and polystyrene tissue culture plate (Figure 7A). The cell viabilities on the sericin hydrogels have no great difference with the cells on polystyrene tissue culture plate (Figure 7B). Furthermore, a large number of proliferative cells were observed on the sericin hydrogel after 3 days seeding, indicating that the sericin hydrogel could promote the growth and proliferation of NIH3T3 cells (Figure 7C). Together, the sericin hydrogel possess excellent cell adhesion and biocompatibility, which can serve as a promising cell carrier for supporting cell survival and proliferation.

## 3. Conclusions

A sterile sericin hydrogel with excellent physicochemical properties and biocompatibility was successfully fabricated in this study. Prior to our study, various attempts were reported for fabricating pure sericin hydrogels based on sericin isolated from silkworm cocoons or silkworm bodies [2,8,25,26,27]. Compared with these sericin hydrogels, our sericin hydrogel system has several advantages: (1) A sterile sericin hydrogel with injectability, good elasticity, and robust behaviors can be fabricated directly. (2) This hydrogel system breaks the previous idea that sericin extracted by high temperature and pressure is difficult to cross-link directly into a stable hydrogel. (3) This hydrogel system retained the small molecular bioactivity polypeptides of silkworm cocoons. Sericin in silkworm cocoons is comprised of a variety of polypeptides with different molecular weights. Many small polypeptides would be discarded inevitably during extracting sericin process at dialysis. (4) The hydrogel production process is simple and efficient. Besides, the gelation time can be controlled by adjusting sericin concentrations. The hydrogel also owns the satisfactory performance of swelling properties, high porosity, sustained drug release, and promoting cell adhesion and proliferation. The new sericin hydrogel system in this study has guiding significance for further expanding and enriching sericin hydrogel applications. 

## 4. Materials and Methods

### 4.1. Materials

The fresh cocoons were produced by *180 Nd-s* fibroin-deficient mutant silkworms that were preserved in our lab. Genipin (purity ≥ 98%) was obtained from Linchuan Zhixin Biotechnology Co., Ltd. (Wuzhou, China). Ninhydrin and horseradish peroxidase (HRP) were supplied by BBI life sciences (Shanghai, China).

### 4.2. Extraction of Sericin Solution

Sericin was isolated from *180 Nd-s* cocoons in the light of our earlier established protocol [26]. Briefly, the cocoons were powdered, and then the powder was thoroughly mixed with ultrapure water. The mixture underwent treatment at 121 °C for 30 min. The obtained soup was centrifuged at 5000 rpm for 5 min to collect the supernatant sericin solution. The concentration of sericin solution was determined by the oven drying method, and the molecular weight distribution of sericin was detected by SDS-page method.

### 4.3. Free Amino Group Content Detection

The quantification of free amino groups in the sericin was determined by ninhydrin colorimetry method according to the previous report with mild modifications [42]. Firstly, glycine, a reference standard, was dissolved and diluted into different concentrations (0.01 mg/mL, 0.02 mg/mL, 0.03 mg/mL, 0.04 mg/mL, and 0.05 mg/mL) of solution using ultra-pure water. The mixture of glycine solution, ninhydrin ethanol solution (2%, *w*/*v*,) and PBS (pH 6.8) were prepared at a constant volume ratio of 1:2:2. Then the mixture was bathed in a hot water at 100 °C for 15 min. After that, the samples were taken out and cooled at room temperature. The absorbance value of samples at 570 nm was performed on a spectrophotometer. A linear standard curve was drawn, plotting the average absorbance values obtained for the mixture triplicate values against the standard glycine concentration. Then the glycine solution was replaced by sericin solution in the above reaction system and the absorbance values of sericin solution samples were obtained. At last, these absorbance values were brought into standard curve equation and then the free amino group content in the sericin solution can be worked out.

### 4.4. Amino Acid Analysis

The amino acid composition of sericin was detected by using high-performance liquid chromatography (HPLC). Briefly, 1 g freeze-dried sericin sample was hydrolyzed by 20 mL HCl (6 M) at 110 °C for 24 h in a blast drying oven. The hydrolyzed sample was transferred into a colorimetric tube and diluted with deionized water to 25 mL when its temperature drops to 25 °C. Next, 1 mL clear solution was collected and blow-dried in the water bath at 85 °C. After that, 1 mL water was added and blow-dried again. The 10 mL HCl (0.02 M) was added and shaken well. Then, 500 μL solution was taken out and mixed with 250 μL phenyl isothiocyanate acetonitrile (0.1 M) and 250 μL triethylamine acetonitrile (1 M). 1 h later, 2 mL n-hexane was added, and mixed well. The reacted sample was then placed statically for a period. The lower solution was tested on a high-performance liquid chromatograph (Agilent 1260, Agilent Technologies, Inc., Palo Alto, USA) after it was filtered through a 0.45 μm organic membrane filter.

### 4.5. Preparation of Sterile Robust Sericin Hydrogel

Genipin was diluted with ultra-water and filtered with a 0.22 μm filter membrane. Then the genipin solution was dried in a sterile environment at 25 °C. The dried genipin was then added into sericin solution according to a mole ratio of genipin to free amino groups in the mixture was 1:2. Following by that, the mixture was mixed thoroughly and formed into a hydrogel in a sterile environment at 25 °C. The hydrogel was defined as n% sericin hydrogel in terms of the concentration of sericin solution in the reaction buffer for gelation.

### 4.6. Gelation Time of Sericin Hydrogel

Gelation time was performed as a previously described protocol [8]. The sericin solution and genipin solution were mixed fully, followed by dropping them into a glass vial. The gelation time was the period between time points of beginning mixing and the mixture became viscous, preventing it from flowing down the vertical wall of the vial. 

### 4.7. Scanning Electron Microscopy (SEM)

The samples were frozen with liquid nitrogen following by dried using a vacuum drying equipment (FD-1A-80, Beijing Boyikang Experiment Instrument Co., Ltd., Beijing, China) at −80 °C. The freeze-dried specimens were carried out on a scanning electron microscopy (JSM-IT300, JEOL Ltd., Tokyo, Japan) and their pore sizes were analyzed using Image J 1.8.0 software.

### 4.8. Porosity Analyses

A frequently used liquid displacement method [26] was employed to detect the porosity of the 8% sericin hydrogel samples. Firstly, the lyophilized sample was put into and covered with a known volume (VK) of ultra-pure water for an hour. The total volume of water containing hydrogel was denoted as VT. Then, the hydrogel sample was taken out and volume of the residual water was recorded as VR. The porosity of the lyophilized hydrogel sample was then calculated using the following equation:

$$\mathrm{porosity}\;\left(\%\right)=\frac{\mathrm{VK}-\mathrm{VR}}{\mathrm{VT}-\mathrm{VR}}\times 100\%$$

### 4.9. Evaluation of Swelling Behaviors and Degradation Dynamics

The swelling behavior of the 8% sericin hydrogel was assessed by a gravimetric method [43]. Lyophilized hydrogels with known weight were submerged in phosphate-buffered saline (PBS) with different pH (pH 3, pH 7.4, and pH 11) at 37 °C. The hydrogel samples were taken out and weighted at predetermined intervals. The swelling ratios of the lyophilized hydrogel scaffolds were calculated by:

$$\mathrm{Swelling}\left(\%\right)=\frac{\mathrm{My}-\mathrm{Mx}}{\mathrm{Mx}}\times 100\%$$

where Mx and My are represent the dry weight and swollen weight of the scaffolds.

The in vitro degradation behavior of 8% sericin hydrogel was tested by a gravimetric method. Briefly, the wet weight (M_wa)_ and dry weight (M_db_) of three randomly chosen hydrogel samples were recorded. Then other dried hydrogels were soaked in PBS (pH 7.4) at 37 °C and the PBS was refreshed every 24 h. The hydrogel samples were extracted, washed, and dried at scheduled time points. The degradation ratios of hydrogels were worked out by:

$$\mathrm{Deradation}=\frac{M0\times R-Md}{M0\times R}$$

where, M0, Md and R represent the initial sample’ wet weight of, the sample’ dry weight, the ratio of M_db_ and M_wa_, and return.

The in vivo degradation behavior of sericin hydrogel was checked refering to a method in previous report [44]. Female C57BL/6 mice (8 weeks old) were first anesthetized by intraperitoneal administration of 10% chloral hydrate (5 μL/g·BW). Next, 8% hydrogel samples (0.2 g per sample) were placed subcutaneously in mice. According to the pre-set time points, the mice were euthanized, followed by resecting the implanted hydrogels and washing carefully with ultra-pure water. Then, the resected samples were dried for further analysis. All the animal experiments were approved and guided by the Ethics Committee of Jiangsu University of Science & Technology (Zhenjiang, China).

### 4.10. The Release of Drug from Sericin Hydrogels

Horseradish peroxidase (HRP), utilized as a model drug, was employed to explore controlled release behavior of the sericin hydrogels. HRP-contained hydrogel samples were fabricated by mixing HRP (2 mg/mL) with sericin solution (8%, *w*/*v*) with genipin at a volume ratio of 1:175. The samples were submerged in fresh PBS (pH 7.4) and kept in a constant temperature incubator at 37 °C. The content of HRP in the PBS buffer was detected by ELISA kit (Thermo Fisher, Shanghai, China).

### 4.11. Fourier Transform Infrared (FTIR) Spectroscopy and X-ray Diffraction

The FTIR spectra of the sericin dried hydrogels were carried out on a Fourier transform infrared spectroscopy (Nexus, Thermal Nicolet, USA) and then analyzed by OPUS 5.5 software (Bruker Optics, GmbH, Frankfurt, Germany). The X-ray diffraction (XRD) scatter of the hydrogel specimens was performed on a D8-Advance X-ray diffractometer (Bruker, Mannheim, Germany).

### 4.12. Mechanical Analysis

The mechanical properties of hydrogels were performed on universal testing machines. Cylindrical hydrogel samples (diameter 8-mm, height 7-mm) and dumbbell shapes (gauge length 15 mm, width 10 mm) were formed in different modes. Then, the former samples were employed for stress-strain analysis and the latter were used for tension test. All the tests were carried out at a constant velocity of 5 mm/min.

### 4.13. Syringe-Injectable Property

The 8% sericin hydrogel was formed in a syringe. Then, the injectability of the sericin hydrogel was analyzed by a syringe with a 16 G needle. 

### 4.14. In Vitro Cell Test

Cells were cultured on the sericin hydrogels formed in the cell-culture plates and the cell-culture plates without hydrogel set as controls. Briefly, the 8% hydrogel samples gelled in 24-well or 96-well culture clusters, followed by 5 rinses with fresh PBS (pH 7.4). Subsequently, the hydrogel samples were rinsed 3 times with fresh high glucose DMEM medium containing 10% fetal bovine serum before further use. Mouse embryonic fibroblasts cells (NIH3T3) were cultured with high glucose DMEM medium in a cell incubator (5% CO_2_, 100% humidity, 37 °C). The cell adhesion behavior of sericin hydrogels was determined according to a reported method [2]. NIH3T3 cells were cultured in 24-well cell culture plates with 1 × 10^4^ cells/well. Additionally, the CCK-8 kit (Dojindo, Kumamoto, Japan) was employed to assess the viability of NIH3T3 cells cultured in the 96-well cell culture plates at a density of 4 × 10^3^ cells/well.

### 4.15. Statistical Analysis

All the experiments were repeated three times and the values were presented as mean ± SD. The significance level between groups was produced by one-way analysis of variance (ANOVA) and Turkey’s test. Image J software was used to analyze the microscopic images. *p* < 0.05 was regarded statistically significant.

## Figures and Tables

**Table 1:** Free amino groups and amino acid composition of sericins isolated from wild-type silkworm cocoons (Jingsong A) and fibroin deficient cocoons (*180 Nd-s*).

Free Amino Groups (in mol/kg)/Amino Acids (in Mole %)	Sericin Isolated from Jingsong A	Sericin Isolated from *180 Nd-s*
Free amino groups	0.09	1.05
Serine	32.81	31.93
Aspartic acid	15.89	15.47
Glycine	10.28	9.68
Threonine	9.13	8.86
Glutamic acid	6.27	7.43
Tyrosine	5.03	5.64
Arginine	4.92	4.73
Lysine	3.49	3.62
Alanine	3.26	3.40
Valine	3.15	3.31
Histidine	1.79	1.72
Leucine	1.64	1.70
Isoleucine	0.87	0.87
Proline	0.73	0.81
Phenylalanine	0.63	0.71
Methionine	0.11	0.11
Cystine	not detected	not detected

**Table 2:** The pore size, porosity and pore wall thickness of the different lyophilized sericin hydrogel scaffolds were frozen at −196 °C.

Hydrogels	4% Sericin Hydrogel	8% Sericin Hydrogel	10% Sericin Hydrogel	12% Sericin Hydrogel
Pore size (μm)	4.9 ± 0.6	6.0 ± 1.9	6.7 ± 0.8	7.1 ± 1.5
Porosity (%)	79 ± 5	70 ± 4	67 ± 1	66 ± 3
Pore wall thickness (μm)	0.31 ± 0.1	0.68 ± 0.2	0.96 ± 0.3	1.39 ± 0.4

**Table 3:** The secondary structure compositions of sericin protein before and after gel formation.

Secondary Structure		A-Helix	Random Coil	β-Sheet	β-Turn
Ratio (%)	Sericin powder	21.95	30.50	25.74	21.81
	Sericin hydrogel	25.74	26.46	32.85	14.95

## Data Availability

The data presented in this study are openly available in article.

## Supplementary Materials

The following supporting information can be downloaded at: https://www.mdpi.com/article/10.3390/gels9120948/s1, Videos S1–S4 (used for demonstrating elasticity, robustness and injectability of the hydrogels).
